# Research Letter: Using participatory approach to facilitate engagement of people with lived experience of schizophrenia in research: A co-designed Participant Information and Consent Form

**DOI:** 10.1177/00048674251388547

**Published:** 2025-11-20

**Authors:** Urska Arnautovska, Rebecca Soole, Nicole Korman, Andrea Baker, Dan Siskind

**Affiliations:** 1 Faculty of Medicine, University of Queensland, Brisbane, QLD, Australia; 2 Metro South Addiction and Mental Health Services, Brisbane, QLD, Australia; 3 Queensland Centre for Mental Health Research, Brisbane, QLD, Australia

## Introduction

Ensuring research participants understand the purpose, methods, risks, benefits and alternatives to participating is critical for ethical and regulatory compliance (Bhutta, 2004). However, Participant Information and Consent Forms (PICFs) are often criticised for hindering valid consent due to their length, complexity and use of scientific jargon, requiring substantial attention and time to comprehend. In response, simplified, participant-friendly PICF versions have recently been developed to improve participants’ accessibility to clinical trials. A noteworthy example is the InFORMed PICF template, developed by Clinical Trials:Impact & Quality (CT:IQ) initiative through a collaborative process with diverse stakeholders, for use by the Australian medical research sector (CT:IQ, 2025). More pictorial-based PICF have also been used outside Australia; examples include the REMAP-CAP, PANORAMIC, ECLS and adAPT trials. The challenges of obtaining ethically and legally robust consent are heightened in research with vulnerable populations, such as individuals living with schizophrenia, who often experience cognitive and motivational difficulties – including reduced attention, executive functioning and working memory – that affect their ability to process trial information and engage in consent discussions. Recruitment challenges, however, go beyond participant factors, including organisational system challenges, identifying eligible participants and engaging clinicians to assist with recruiting consumers. Such multi-level challenges can be addressed through shared decision-making and collaborative problem solving (Jørgensen et al., 2014). Given the consenting challenges related to people living with SMI such as schizophrenia, we adopted co-design methodology to re-design the traditional, lengthy PICF documents to better suit the preferences and needs of the target population.

## Methods

We conducted two face-to-face participatory co-design sessions with five residents of a metropolitan community care unit (CCU) living with schizophrenia. The participants were part of an established Consumer Reference Group and were reimbursed for their time participating in each session with an AUD $50 gift voucher. Sessions were approximately 45–60 minutes long and facilitated by two clinical academics (U.A. and N.K.) who took notes during the sessions to capture participants’ preferences and needs related to PICF.

The aim was to redesign the PICF used in our research team’s clinical trials, to improve user engagement and comprehension, while maintaining ethical compliance and meaningful informed consent. In the first session, the consumer representatives reviewed the original 15-page PICF in paper format and provided feedback on its length, language, structure, visual layout and suggestions for improvements. Based on their feedback, a new visual PICF was developed. In the second session, consumer representatives reviewed the revised draft PICF, providing additional edits and final approval.

While participants provided their informed consent to be involved in the co-design sessions, ethical approval for the co-production of the PICF was not required. Involvement of consumers in the early stages of research, prior to a research study undergoing a full review by the ethics committee, is part of best practice.

## Results

The co-design sessions revealed a clear preference for a shorter, more visually engaging PICF. Consumer representatives highlighted the need for aesthetically appealing visual prompts to serve as anchors for discussion and comprehension. This format was deemed more user-friendly, motivating and less overwhelming, especially for individuals first learning about the research.

**Figure 1.**
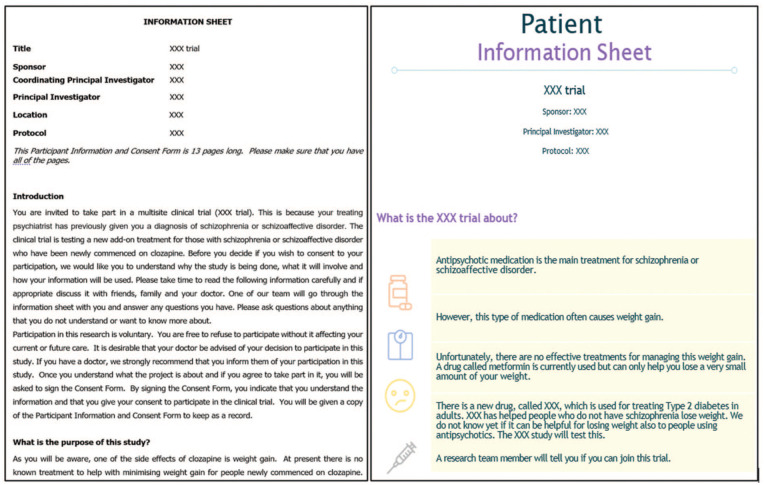
Page 1 of the original (left) and co-designed visual PICF (right).

Participants valued being asked for input and appreciated seeing their suggestions reflected in the final product. Contributing in this way was described as meaningful and affirming. They also recognised the need for a longer, more detailed PICF for those seeking in-depth information. Consequently, our final design included two formats: a short, icon-based PICF (5 pages; Supplemental Appendix 1) for initial conversations and a comprehensive, text-based PICF (13 pages) for deeper engagement. To link the two, participants suggested the use of consistent visual icons (Figure 1) to support a seamless transition between both formats.

Additional refinements included a question-and-answer format for each section, allowing readers to quickly locate relevant information. Participants also provided valuable feedback on identifying jargon that could be simplified to improve accessibility and language modifications to emphasise autonomy.

The newly designed short and long-form PICFs received ethical clearance for use in our randomised, double-blind, adaptive trial of tirzepatide against placebo in people with schizophrenia/schizoaffective disorder and overweight/obesity (HREC/2023/QMS/103997).

## Discussion

By adopting a co-design approach, we were able to work in close partnership with people with lived experience of schizophrenia to develop a more accessible and meaningful PICF. Their insights helped ensure the document supported engagement, comprehension and ethically informed consent, particularly for people with cognitive and motivational challenges. Beyond practical feedback, participants’ involvement in shaping research materials and seeing those changes implemented contributed to their recovery process, affirmed their expertise and value, and reflected the co-design principles of research needing to be designed *with*, rather than for, the individuals that it is intended to serve. It also provided an opportunity to challenge both public- (e.g. misconceptions around schizophrenia, fuelling fear and exclusion) and self-stigma (e.g. internalised negative beliefs about one’s self-esteem and self-efficacy) and shift narratives – both in clinical research and in broader systems of care.

Consumer involvement in health research is recognised nationally (NHRMC, 2016) and internationally (Nilsen et al., 2006) as critical in conducting person-centred, ethical and effective research. National guidelines and frameworks now encourage meaningful consumer engagement across all stages of the research process and numerous resources have been developed to support this (NHRMC, 2016). In addition, to further improve participant engagement in clinical trials, eConsent options should be explored, taking into account key considerations for eConsent – a detailed overview of these was conducted by the Australian Medical Research Future Fund-funded CT:IQ initiative (CT:IQ, 2019), however, their implementation is still in infancy.

Our findings contribute to a growing body of evidence that traditional PICFs can unintentionally act as barriers to valid consent, particularly in populations with cognitive challenges, and offer a practical template for developing accessible consent materials in trials where attention and comprehension may be impacted. The insights gained have already begun to shape research practices within our team and globally (Soole et al., 2023), further reinforcing the idea that integrating lived experience leads to more feasible, relevant and inclusive research.

Our work co-designing the PICF using qualitative methods aligns with research in other domains adopting complex adaptive platform trial designs (Symons et al., 2022). By incorporating lived experience into the consent process, these trials are more likely to be adopted and embedded within routine clinical practice. Such lived experience-informed PICF templates may also be more readily adopted by institutional and health service HRECs due to their improved acceptability and suitability, potentially leading to more informed and robust consent. However, each trial will require tailored adjustments to the visual PICF template, with clear contextual explanations provided to the HREC members.

The outcomes of this study underscore the importance of customising PICFs to meet the diverse needs of potential participants. By integrating the perspectives of those with lived experience, we enhanced the accessibility and relevance of these materials, promoting informed decision-making, and strengthening the ethical foundations of schizophrenia research, and, importantly, protecting the basic rights of trial participants.

## Conclusion

Tailoring PICFs to the unique needs of target individuals is a critical step in promoting ethical, informed participation in research. Through co-design, we developed materials that support autonomy, reduce cognitive burden, and encourage meaningful and equitable engagement with people who have often been marginalised in research contexts.

Such participatory co-design processes contribute to reducing stigma and ensuring that people affected by SMI can participate meaningfully in research that affects them. Co-design should be considered best-practice when developing research materials for vulnerable populations and implemented alongside established guidelines for involving people with lived experience and their families in mental health research (Hawke et al., 2025). This approach not only improves access to research but also fosters a more inclusive and respectful environment for participants and lived experience collaborators.

If you are interested in learning more about our co-designed PICFs or considering them for your research, please feel free to adopt for your research purposes a Word version of the co-designed PICF provided in Supplemental Appendix 1 or reach out to us at u.arnautovska@uq.edu.au. If you adapt our PICF for your trials, we would appreciate our design process being cited.

## Supplemental Material

sj-docx-1-anp-10.1177_00048674251388547 – Supplemental material for Research Letter: Using participatory approach to facilitate engagement of people with lived experience of schizophrenia in research: A co-designed Participant Information and Consent Form Supplemental material, sj-docx-1-anp-10.1177_00048674251388547 for Research Letter: Using participatory approach to facilitate engagement of people with lived experience of schizophrenia in research: A co-designed Participant Information and Consent Form by Urska Arnautovska, Rebecca Soole, Nicole Korman, Andrea Baker and Dan Siskind in Australian & New Zealand Journal of Psychiatry
