# Supplementary material for: Research Letter: Using participatory approach to facilitate engagement of people with lived experience of schizophrenia in research: A co-designed Participant Information and Consent Form
Source: Aust N Z J Psychiatry. 2025 Nov 20;60(6):599–601. doi: 10.1177/00048674251388547 (PMC13191050; doi:10.1177/00048674251388547)
Supplement: sj-docx-1-anp-10.1177_00048674251388547 – Supplemental material for Research Letter: Using participatory approach to facilitate engagement of people with lived experience of schizophrenia in research: A co-designed Participant Information and Consent Form [file sj-docx-1-anp-10.1177_00048674251388547.docx]

Patient


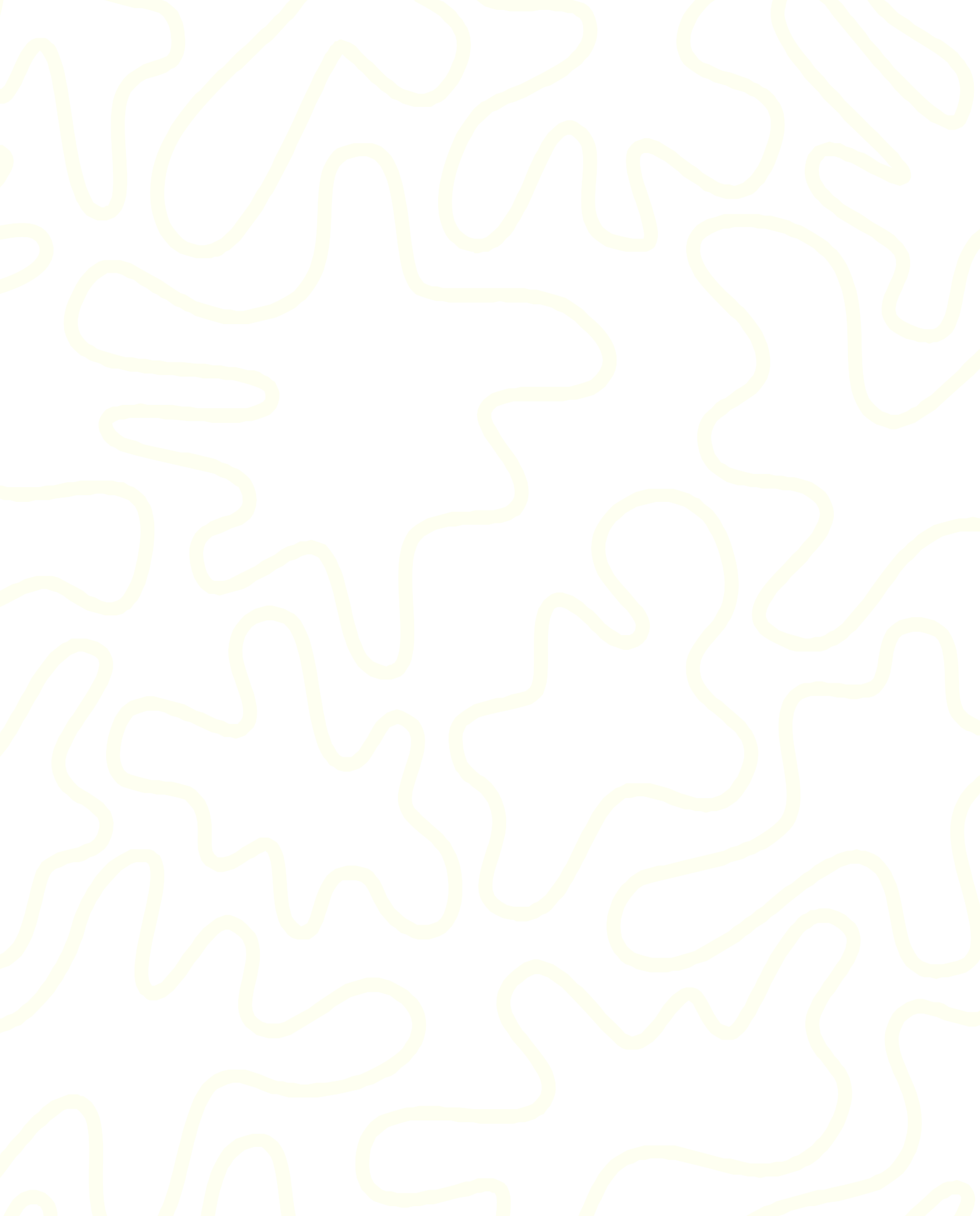

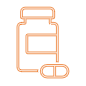

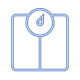

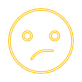

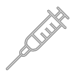


Information Sheet

XXX trial

Sponsor: XXX

Principal Investigator: XXX

Protocol: XXX

.

**What is the XXX trial about?**

| Antipsychotic medication is the main treatment for schizophrenia or schizoaffective disorder. |
| --- |
| However, this type of medication often causes weight gain. |
| Unfortunately, there are no effective treatments for managing this weight gain. A drug called metformin is currently used but can only help you lose a very small amount of your weight. |
| There is a new drug, called XXX, which is used for treating Type 2 diabetes in adults. XXX has helped people who do not have schizophrenia lose weight. We do not know yet if it can be helpful for losing weight also to people using antipsychotics. The XXX study will test this.  A research team member will tell you if you can join this trial. |

Visual Participant Information Sheet Version/Date; V1.0, Date Page 1


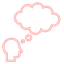

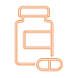
**Who can take part?**

| **Eligibility** | Anybody aged 18-64 years AND  Diagnosed with schizophrenia or schizoaffective disorder AND  Taking antipsychotic medication for at least 12 weeks WITH  overweight/obesity |
| --- | --- |
| **Voluntary** | Your participation is entirely voluntary - your care will not be affected, whether you choose to take part or not. |


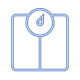

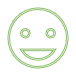
**What will happen if I take part?**


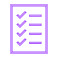
**Screening**


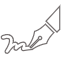
**Consent**


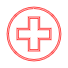
 **Treatment**


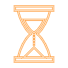
**Duration**

We will ask you to take part in a screening process (lasting about 1 hour). This will involve questions about your mental illness. You can have a friend, family member, or peer support worker with you for this if you like.

When you are ready, we will ask you to sign a Consent Form to show that you agree to participate and that you understand all the information.

If you agree, you will get a weekly injection of either XXX or placebo. The XXX/placebo dose will begin at XXmg and increase gradually over 13 weeks, as follows:

**Injection**

Dose

**Week 1**

2.5mg (0.5mls) weekly

**Week 5**

5.0mg (0.5mls) weekly

**Week 9**

7.5mg (0.5mls) weekly

**Week 13**

10mg (0.5mls) weekly

[Dosing table goes here]

You will stay on the highest tolerated dose for the rest of the trial.

The trial runs for 24 weeks.


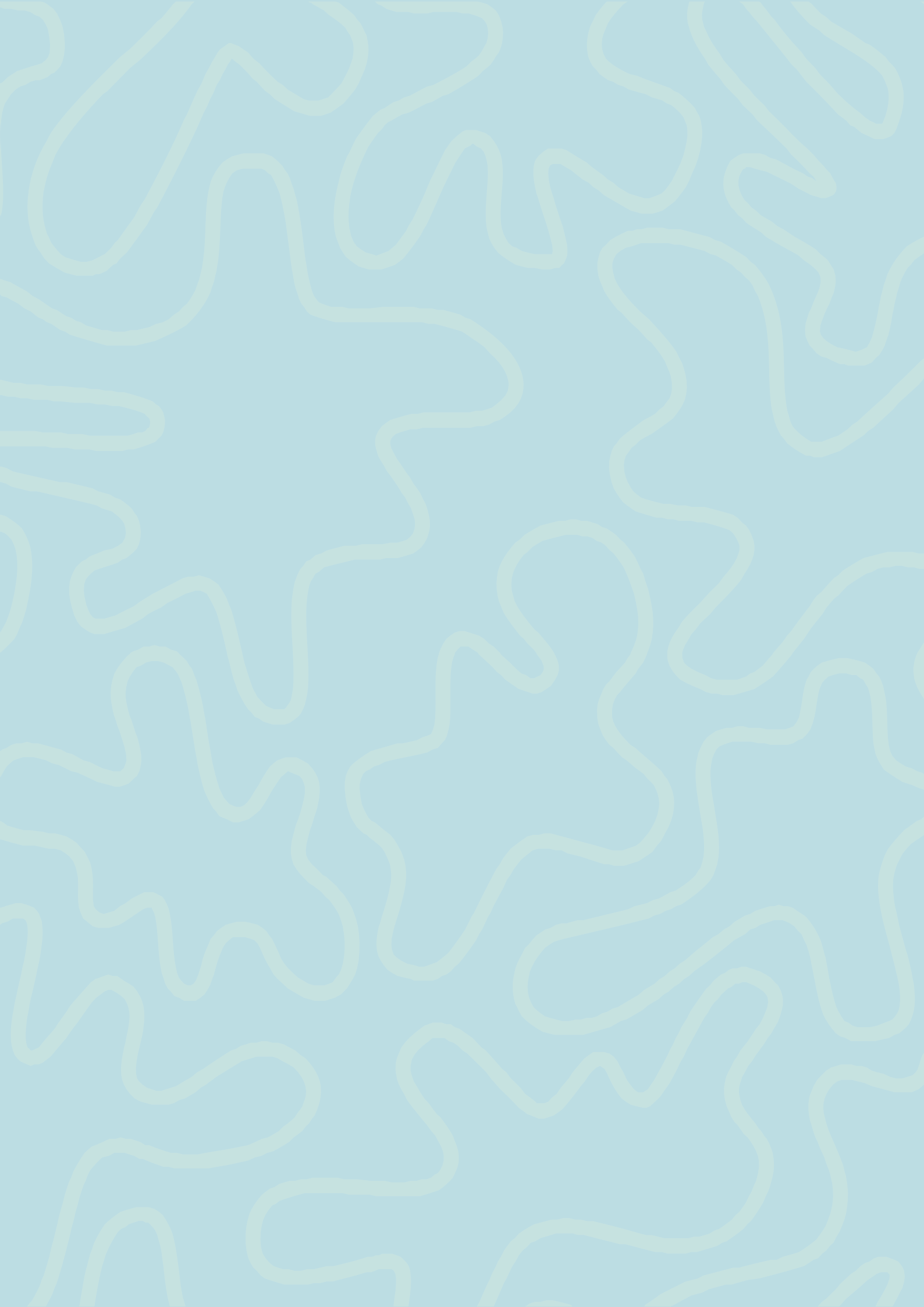


Visual Participant Information Sheet Version/Date; V1.0 Date Page 2


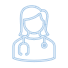

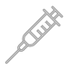
**What assessments will I do?**


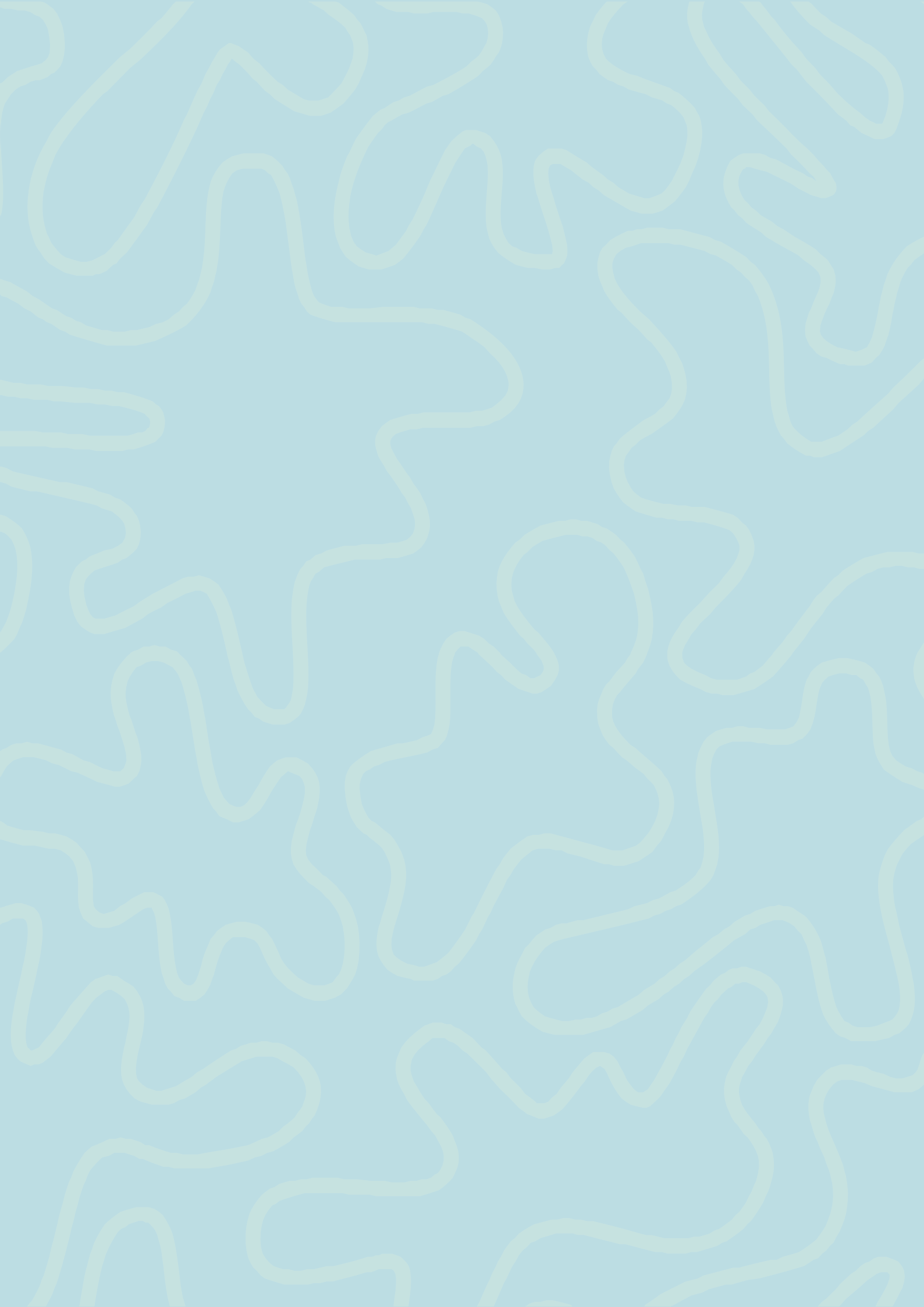


| **Contact with the clinical team** | You'll meet weekly with the trial team for your injection. The team will check your health measures like blood pressure, height, weight, and waist circumference, taking about 15 minutes.  At baseline, week 12 and 24, there will be more detailed assessments lasting about 90 minutes. These assessments cover mental and physical health, diet, exercise, bowel habits, cognitive abilities, and alcohol use. |
| --- | --- |
| **Blood sample** | You'll give three blood samples during the trial at baseline and week 24. This helps measure physical indicators of weight change, like fats in your blood. |
| **Optional Dual Energy X‐Ray Absorptiometry (DEXA)** | You'll be asked to have two DEXA scans: one at the beginning and another at week 24. DEXA measures body composition (muscle and fat) with low radiation. The scan is quick (about 10-20 minutes), painless, and you'll lie on a table while a scanning arm passes over your body. |
| **Unexpected findings in DEXA** | Unexpected findings in the scans are rare, and the scans are not for diagnosis. If an unusual finding occurs during your DEXA scan, the radiologist will report it to the Senior Medical Officer. Your identity is only shared if further tests are needed. |


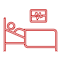

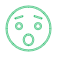

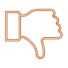
**What is the risk of taking part?**

| **Risks** | Participating in this trial carries potential risks, including: Discussing mental health may be distressing; if uncomfortable, we'll stop the conversation and provide support.  Clinical assessments may take time; if tiring, you can request breaks or split them into sessions.  Weight and waist measurements will be done respectfully but might evoke uncomfortable feelings.  Injections or blood tests may cause slight discomfort, bruising, inflammation, or minor infection, usually infrequent and minor. Medication may have side effects, with varying severity.  Discuss any concerns with the trial team during your visits. DEXA scans, though generally safe, lying flat may cause discomfort; inform the radiographer if needed. |
| --- | --- |
| **Potential side effects of XXX** | You may experience nausea, vomiting, diarrhea, swelling of nasal passages, dizziness, fatigue, increased heart rate, decreased weight and appetite, and hypersensitivity. If you notice any new or unusual symptoms, inform the trial team immediately.  To participate, you must not be pregnant, breastfeeding, or trying to conceive. If you become pregnant, notify the trial team promptly. Your safety is our top priority. |


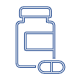
Visual Participant Information Sheet Version/Date; V1.0, Date Page 3

**What is the advantage of taking part?**


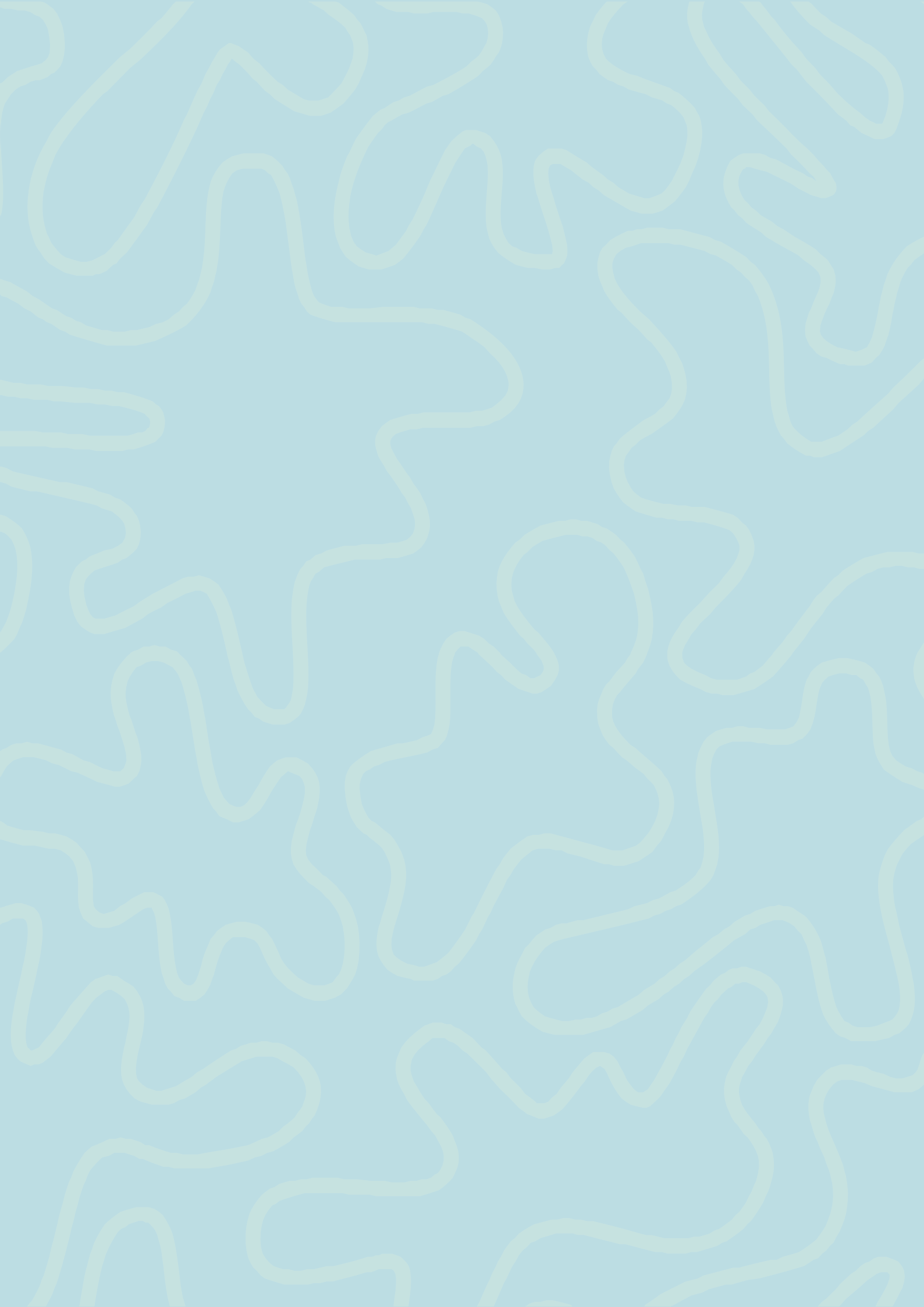


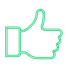


**Benefits**

We can't guarantee personal benefits from this trial. Your participation is vital in assessing how well XXX works as an 'add-on' treatment for antipsychotic-related weight gain. Specific benefits are uncertain, but many participants find the tasks engaging and satisfying.

**Is there any cost involved in taking part?**

| **No cost** | There is no cost involved - all medication, tests, and medical care related to the trial are provided for free. |
| --- | --- |
| **Thanks** | To thank you for your time, you will receive gift cards: at baseline, weeks 12, and 24, a $50 gift card; for each four-weekly visit, a $20 gift card; and  if you choose to do the DEXA scan, an extra $30 at baseline and week 24. |


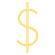

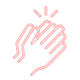

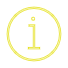
**What could change?**

| **New information becomes available** | During clinical trials, new information about the treatment may arise such as updates on safety of XXX. If this happens, we may need to discontinue your involvement for your well-being.  If new interventions become available, we will contact you to discuss options of participating. |
| --- | --- |
| **Stopping the clinical trial** | This trial could unexpectedly stop for reasons like unacceptable side effects, the drug not performing well, or drug supply issues. The investigator may also end your participation for reasons like injury, pregnancy, a medical condition posing risks, or not following medication instructions. |


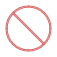
**What if I want to stop the trial?**


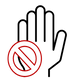


**Changed my mind**

All research is voluntary - you can choose to withdraw at any time without impacting your current or future care. If you decide to withdraw, just let the trial team know.

**What happens at the end of the trial?**


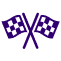


**End of trial**

De-identified data will be analysed and the results shared in scientific journals and events. You will receive a summary of results including your group assignment once all participants complete the trial.

Visual Participant Information Sheet Version/Date; V1.0, Date Page 4


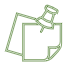
**What happens to my information?**


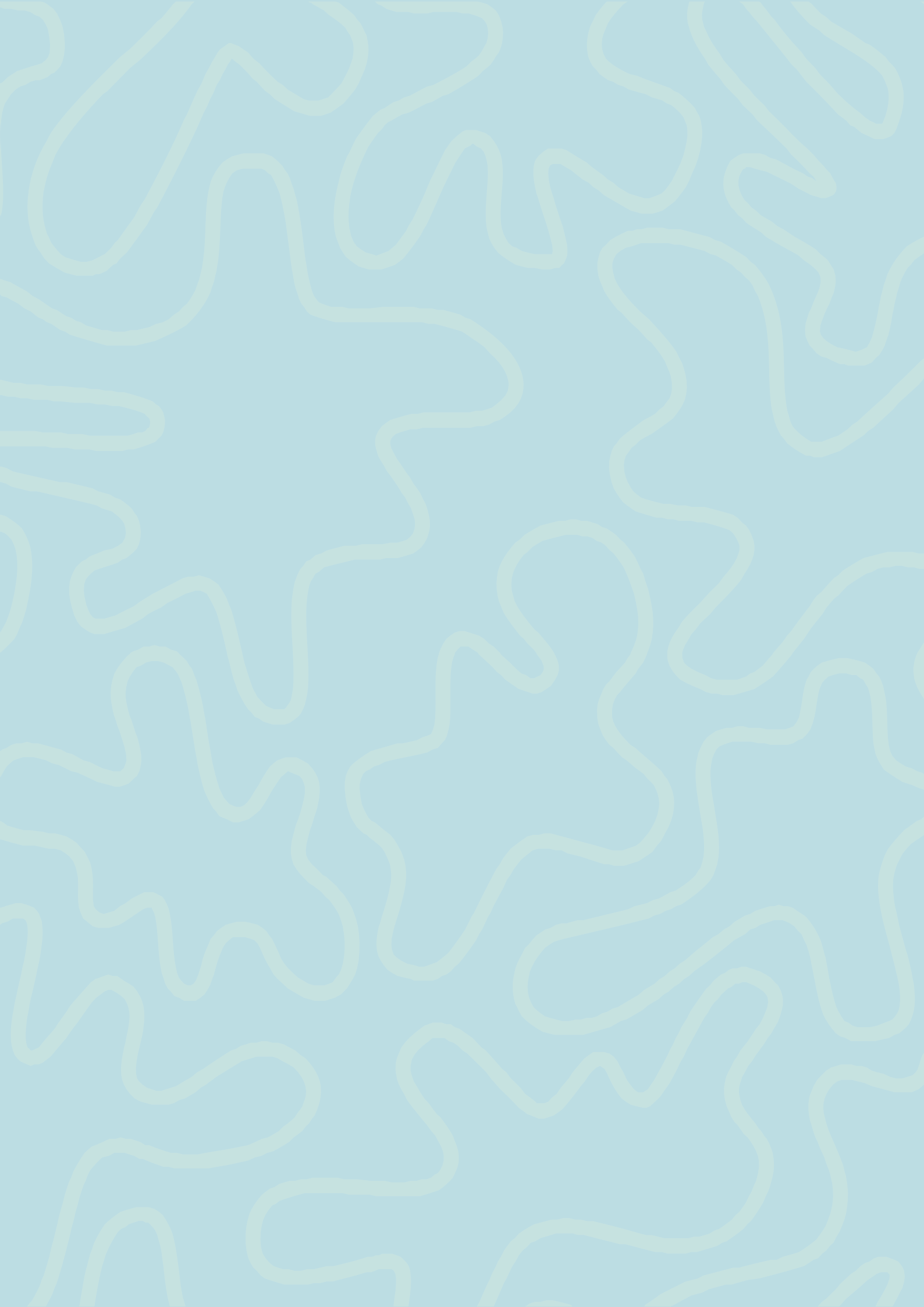


| **Identifying information** | Any information identifying you in this trial will be kept confidential and used only for the trial, disclosed with your permission, or as required by law. |
| --- | --- |
| **Accessing my information** | As per Australian and/or Queensland privacy laws, you can ask to see or correct the information collected about you by contacting the research team member listed in the document's question section. |


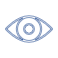

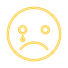

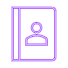
**What if there is a problem?**

| **Injury** | If you suffer an injury because of participating in this trial, hospital care and treatment will be provided at no extra cost if you elect to be treated as a public patient at a public health service. |
| --- | --- |
| **Ethical Guidelines and Independent Contact** | This clinical trial has been approved by Metro South Human Research Ethics Committee and local site Governance.  If you have any complaints about any aspect of this trial, you can contact the HREC Coordinator, XXX (phone) or [XXX](mailto:msh-ethics@health.qld.gov.au) (email). All complaints will be treated in confidence, investigated fully and you will be informed of the outcome. |


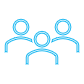
**Who can I contact?**

| **Research team** | Name XXX  Address XXX  Email XXX |
| --- | --- |
| **More questions** | Please read carefully both, the visual Patient Information Consent Form as well as the more detailed Patient Information Consent Form that has been provided to you. You can speak with your friends, family, peer support worker, or doctor if needed. Our team is available to explain everything and answer your questions about anything that you would like to know more about.  **Your wellbeing is our priority.** |


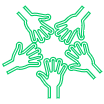
Visual Participant Information Sheet Version/Date; V1.0, Date Page 5
